# Prognostic nomogram for female patients suffering from non-metastatic Her2 positive breast cancer: A SEER-based study

**DOI:** 10.1097/MD.0000000000030922

**Published:** 2022-10-07

**Authors:** Jiangwen Wu, Zhaomin Xie, Yu Xiao, Bingbing Wang, Pengcheng Zhang

**Affiliations:** a Pain Department Cancer Pain Specialty, Xiangyang No.1 People’s Hospital, Hubei University of Medicine, Xiangyang, Hubei Province, P.R. China; b Department of Medical Oncology, The Cancer Hospital of Shantou University Medical College, Shantou, P.R. China; c Guangdong Provincial Key Laboratory for Breast Cancer Diagnosis and Treatment, Cancer Hospital of Shantou University Medical College, Shantou, P.R. China; d Department of Dermatology, The Central Hospital of Xiaogan, Xiaogan Hospital Affiliated to Wuhan University of Science and Technology, Hubei, P.R. China; e Department of Pediatrics, Xiangyang No.1 People’s Hospital, Hubei University of Medicine, Xiangyang, Hubei Province, P.R. China; f Department of Thyroid and Breast Oncology, Xiangyang No.1 People’s Hospital, Hubei University of Medicine, Xiangyang, Hubei Province, P.R. China.

**Keywords:** her2 positive breast cancer, nomogram, SEER, survival

## Abstract

This paper aimed at constructing and validating a novel prognostic nomogram, so that physicians forecast the overall survival (OS) rates of female patients suffering from non-metastatic human epidermal growth element receptor-2 (HER2) positive breast.

Information of primary female her2 positive breast cancer patients without metastasis was obtained from the Surveillance, Epidemiology, and End Results (SEER) database with given inclusion and exclusion standards. Independent variables were obtained greatly by performing univariable and multivariate analyses. Based on those independent predictors, a novel prognostic nomogram was constructed for predicting the survival of those with 3- and 5-year OS. Then, concordance index (C-index), receiver operating characteristic curve (ROC), and calibration plot were adopted for the assessment of the predictive power of the nomogram.

A total of 36,083 eligible patients were classified into a training cohort (n = 25,259) and a verification cohort (n = 10,824) randomly. According to the identification of multivariate analysis, survival was predicted by age at diagnosis, marital status, race, site, T stage, N stage, progesterone receptor (PR) status, estrogen receptor (ER) status, surgery, radiation, and chemotherapy independently. A nomogram was established by applying the training cohort. The nomogram displayed excellent discrimination and performance as indicated by the C-index (0.764, 95% confidence interval: 0.756–0.772), and the 3- and 5-year area under the curve of ROC (AUC) values (0.760 and 0.692 respectively). The calibration plots for forecasting the 3- and 5-year OS were in great agreement.

The OS for female her2 positive breast cancer patients without metastasis was predicted by constructing a nomogram on basis of the SEER database. A precise survival prediction could be offered for each patient.

## 1. Introduction

Surpassing lung cancer, female breast cancer has been the most common cancer with approximately 2.3 million new cases (11.7%) on basis of GLOBOCAN 2020 estimates of cancer incidence and mortality issued by the International Agency for Study of Cancer.^[1]^ In females, breast cancer is the most common cancer and the major cause of death. As a member of the epidermal development factor receptor family of transmembrane receptors, the human epidermal growth factor receptor-2 (HER2) receptor tyrosine kinase exerts important effects on both growth and cancer.^[2,3]^ Amplification and overexpression of the HER2 gene are present in 20% to 25% of human breast cancer and are associated with poor clinical results.^[3,4]^ HER2 protein overexpression is measured by immunohistochemistry (IHC)-based test. A positive HER2 test is defined as IHC3+ or by fluorescence in situ hybridization (FISH) measurement of a HER2 gene copy number of six or more or a HER2/CEP17 ratio of 2.0 or greater.^[4]^ The majority of patients with HER2-positive breast cancer receive surgery and postoperative chemotherapy, while an increasing number also receive neoadjuvant chemotherapy.^[5]^ Before HER2-directed therapies are available, females suffering from HER2-positive breast cancer typically exhibit shorter disease relapse, and grew incidence of metastases, resulting in a worse prognosis than HER2-negative breast cancer.^[6]^ As anti-HER2 therapies are introduced to the treatment of patients suffering from HER2-positive breast cancer, great improvements in survival in both early and advanced settings have been led. Although survival of each patient has improved based on the emergence of these targeted therapies partly, the prognosis can be extremely variable.^[7]^ It’s not completely understood how the tumor characteristics and other patients’ factors influence the treatment benefit and prognosis of HER2-positive breast cancer. By incorporating and illustrating important prognostic factors, physicians used nomograms to accurately estimate the prognosis of patients in time.^[8]^ In this study, the female patients suffering from non-metastatic HER2 positive breast cancer were analyzed on basis of the Surveillance, Epidemiology, and End Results, US = United States (SEER) Program, including the clinicopathological characteristics and prognostic elements. A nomogram was further established and validated to predict the personal 3- and 5-year overall survival (OS) rates of female patients suffering from non-metastatic HER2-positive breast cancer.

## 2. Materials and Methods

### 2.1. Data source

The SEER database is one of the most authoritative databases from the National Cancer Institute of the United States (US) Institutes of Health (https://seer.cancer.gov/). Incidence, prevalence, and mortality information of cancer registries covering about 34.6% of the US population are collected by SEER currently. The identification of data for this research was made from the SEER.

### 2.2. Research population

Population information includes all females suffering from non-metastatic HER2 positive breast cancer and was extracted from the SEER Research Plus Data (18 Regs, Nov Sub 2000–2018) with the SEER*Stat 8.3.9. The given standards for the SEER*Stat software for the identification of patients were shown below: female patients; “Breast” was confined to Site and Morphology (TNM 7/CS v0204 + Schema thru 2017); Originated M phase, American Joint Committee on Cancer (AJCC) M 7th ed (2010–2015) was limited to “M0”; Derived HER2 Record (2010+) was confined to “Positive”; and First malignant primary indicator was limited to “Yes”.

### 2.3. Variables

For each case, SEER provided the data below: age at diagnosis, race, marital status, laterality, site, grade, T stage (AJCC, 7th ed.), N stage (AJCC, 7th ed.), breast cancer subtype, surgery, radiation, chemotherapy, survival months, and vital state. We excluded the following cases: unknown race or marital status, laterality is bilaterally or side unspecified, unknown AJCC T stage, N stage histological grade, estrogen receptor (ER) Status, progesterone receptor (PR) Status. The race category defined “Other” as Pacific Islander or Asian, American Indian/Alaska Native. In the marital status category, “Not married” Included Single, Divorced, Widowed, Separated, Unmarried, or Domestic Partner. The site category defined “Other” as Nipple/Central portion of breast/Axillary tail of breast/Overlapping lesion of breast/Breast, NOS. December 31, 2018, is the cutoff date of follow-up time. The publishing of the TNM stage system (AJCC stage group 7th edition) was performed in 2010, and the same year HER2 status in the SEER database only becomes available. Therefore, the actual date of follow-up for this version of the sub-database is 2010 to 2018.

### 2.4. Nomogram establishment

The eligible patients identified from SEER registries were randomly fallen at a ratio of 7:3 into a training cohort and a verification cohort. A nomogram was established by a training set. Independent prognostic variables were obtained greatly by performing univariable and multivariate analysis. Hazard ratios (HRs) were shown with their 95% confidence intervals (CIs). Based on those independent predictors, novel prognostic nomograms were constructed for predicting the survival of those with 3- and 5-year OS.

### 2.5. Verification of the nomogram

The internal verification and validation of the nomogram were conducted in the training cohort. While the external validation was performed in the verification cohort to evaluate the prediction efficiency. Harrell concordance index (C-index), and the area under the curve of ROC (AUC) were used to evaluate the discrimination of the nomogram. More precise prognostic predictions are indicated by a higher C-index or a higher AUC value.^[8]^ An excellent discriminative capacity between 0.71 and 0.90 is shown by the C-index, while the C-index more than 0.90 displays higher precision. Similarly, the higher the AUC value is, the better the predictive ability of the nomogram will be. The nomogram performance was evaluated by the calibration plot. For a fully calibrated model, the calibration plot shall fall the forecasts at a diagonal 45° line.

### 2.6. Statistical exploration

The comparison of pathological and clinical features of the training and verification cohorts was made with the chi-square as suitable. The Kaplan–Meier approach was adopted to calculate the cumulative survival curves for each patient variable. Univariate and multivariate Cox regression explorations were applied to recognize the significant independent prognostic variables. Due to two-sided P values, values of <.05 were regarded statistically significant. IBM SPSS Statistics 26.0 (SPSS, Inc, Chicago, IL) was adopted to perform univariate and multivariate Cox analyses. R software version 4.0.3 (http://www.R-project.org) was employed to construct the nomogram, receiver operating characteristic curve (ROC), and calibration plots. There were survival and rms in the R package.

## 3. Result

### 3.1. Clinicopathological features of the training and verification sets

Our research investigated 41,497 female HER2 positive breast cancer in total without metastasis cases according to the SEER database. Of these, 5414 patients were excluded because of inadequate data. Our analysis included the 36,083 eligible patients remaining, with 25,259 patients in the training set and 10,824 patients in the verification set. Table 1 presents the clinicopathological features of training and verification sets.

**Table 1:** The demographics and clinical features for female patients suffering from non-metastatic HER2 positive breast cancer in different cohorts.

	Total n = 36,083(%)	Training cohort n = 25,259 (%)	Validation cohort n = 10,824 (%)	*P* value
**Age**				
<40 yr old	3798(10.5)	2651(10.5)	1147(10.6)	.7733
>40 yr old	32,285(89.5)	22,608(89.5)	9677(89.4)	
**Marital status**				
Married	21,686(60.1)	15,212(60.2)	6474(59.8)	.4634
Not married	14,397(39.9)	10,047(39.8)	4350(40.2)	
**Race**				
White	27,321(75.7)	19,094(75.6)	8227(76.0)	.5641
Black	4436(12.3)	3107(12.3)	1329(12.3)	
Other	4326(12.0)	3058(12.1)	1268(11.7)	
**Laterality**				
Left	18,444(51.1)	12,909(51.1)	5535(51.1)	.9586
Right	17,639(48.9)	12,350(48.9)	5289(48.9)	
**Site**				
UOQ	12,141(33.6)	8508(33.7)	3633(33.6)	.7395
LOQ	2927(8.1)	2038(8.1)	889(8.2)	
LIQ	2028(5.6)	1432(5.7)	596(5.5)	
UIQ	3973(11)	2749(10.9)	1224(11.3)	
Other	15,014(41.6)	10,532(41.7)	4482(41.4)	
**Grade**				
Ⅰ	1872(5.2)	1357(5.4)	515(4.8)	.4875
Ⅱ	13075(36.2)	9117(36.1)	3958(36.6)	
Ⅲ	20,948(58.1)	14,656(58.0)	6292(58.1)	
Ⅳ	188(0.5)	129(0.5)	59(0.5)	
**T_stage**				
T0	65(0.2)	40(0.2)	25(0.2)	.1037
T1	17,674(49.0)	12,401(49.1)	5273(48.7)	
T2	13,721(38.0)	9593(38.0)	4128(38.1)	
T3	2895(8.0)	2026(8.0)	869(8.0)	
T4	1728(4.8)	1199(4.7)	529(4.9)	
**N_stage**				
N0	21,586(59.8)	15,121(59.9)	6465(59.7)	.8707
N1	10,455(29.0)	7337(29.0)	3118(29.0)	
N2	2497(6.9)	1783(7.1)	764(7.1)	
N3	1545(4.3)	1068(4.2)	477(4.4)	
**Subtype**				
HR+/HER2+	25,446(70.5)	17,834(70.6)	7612(70.3)	.5938
HR–/HER2+	10637(29.5)	7425(29.4)	3212(29.7)	
**ER**				
Positive	24742(68.6)	17,343(68.7)	7399(68.4)	.5695
Negative	11,341(31.4)	7916(31.3)	3425(31.6)	
**PR**				
Positive	18,959(52.5)	13,270(52.5)	5689(52.6)	.9675
Negative	17,124(47.5)	11,989(47.5)	5135(47.4)	
**Surgery**				
Yes	34,257(94.9)	23,981(94.9)	10,276(94.9)	.9897
No	1826(5.1)	1278(5.1)	548(5.1)	
**Radiation**				
Yes	17,369(48.1)	12,122(48.0)	5247(48.5)	.3983
No	18,714(51.9)	13,137(52.0)	5577(51.5)	
**Chemotherapy**				
Yes	27,172(75.3)	19,023(75.3)	8149(75.3)	.9592
No	8911(24.7)	6236(24.7)	2675(24.7)	

HER2 = human epidermal growth factor receptor-2, HER2+ = human epidermal growth factor receptor-2 positive, HR– = hormone-receptor-negative, HR+ = hormone-receptor-positive, LIQ = lower inner quadrant, LOQ = lower outer quadrant, UOQ = upper outer quadrant, UIQ = upper inner quadrant.

### 3.2. Separate prognostic elements in the training set and establishment of the nomogram

**Figure 1.**
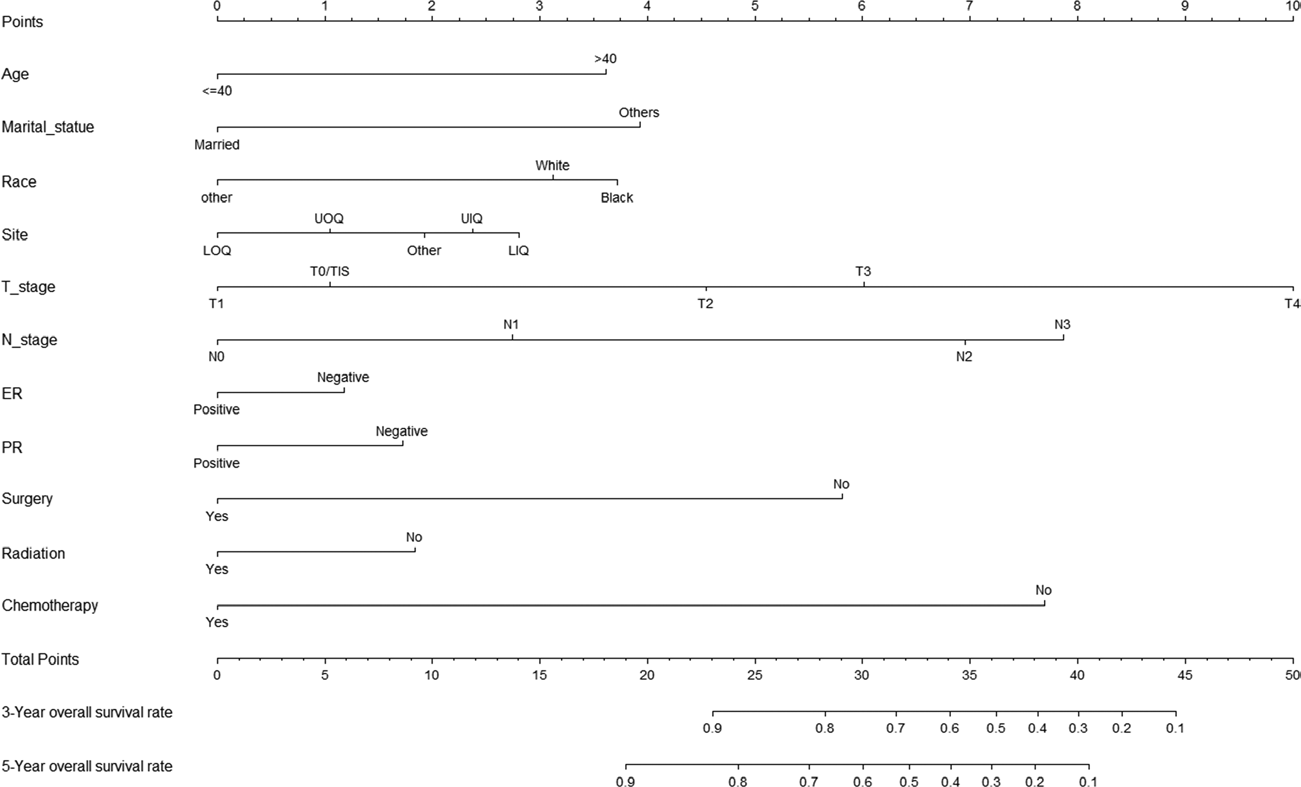
Nomogram for predicting 3- and 5- year OS rates of female patients suffering non-metastatic her2 positive breast cancer.

The nomogram was established with a training set. Table 2 displays univariate and multivariate explorations of hidden predictors for the OS. Age at diagnosis, marital status, race, site, grade, T stage, N stage, breast cancer sub-type, PR status, ER status, surgery, radiation, and chemotherapy, were critically related to risk elements for the OS in the univariate exploration. Hence, multivariate exploration included the mentioned significant risk elements. According to the identification of multivariate analysis, age at diagnosis, marital status, race, site, T stage, N stage, PR status, ER status, surgery, radiation, and chemotherapy could predict survival (Table 2) independently. The nomogram for 3-, and 5-year OS (Fig. 1) was built with independent elements.

**Table 2:** Univariable and multivariate cox analysis for female patients suffering from non-metastatic HER2 positive breast cancer.

Variable	Univariable		Multivariable	
HRs(95%CI)	*P* value	HRs (95%CI)	*P* value
**Factors selected**				
**Age**				
<40 yrs old	Reference	NA	Reference	NA
>40 yrs old	1.84(1.60–2.11)	<.001	1.68(1.46–1.94)	<.001
**Marital status**				
Married	Reference	NA	Reference	NA
Not married	2.22(2.07–2.38)	<.001	1.94(1.63–1.88)	<.001
**Race**				
White	Reference	NA	Reference	NA
Black	1.30(1.18–1.43)	<.001	1.09(0.99–1.20)	.087
Other	0.65(0.57–0.74)	<.001	0.64(0.56–0.72)	<.001
**Laterality**				
Left	Reference	NA	Reference	NA
Right	0.98(0.91–1.05)	.483	0.97(0.91–1.04)	.407
**Site**				
UOQ	Reference	NA	Reference	NA
LOQ	0.84(0.72–0.98)	<.05	0.86(0.74–1.00)	<.05
LIQ	1.10(0.94–1.29)	.21	1.28(1.10–1.50)	<.01
UIQ	1.06(0.94–1.20)	.36	1.21(10.7–1.37)	<.01
Other	1.29(1.19–1.40)	<.001	1.13(1.05–1.23)	<.01
**Grade**				
Ⅰ	Reference	NA	Reference	NA
Ⅱ	1.10(0.93–1.32)	.26	0.98(0.82–1.17)	.843
Ⅲ	1.41(1.19–1.67)	<.001	1.09(0.92–1.30)	.322
Ⅳ	1.56(1.01–2.43)	<.05	0.99(0.63–1.54)	.961
**T_stage**				
T0	Reference	NA	Reference	NA
T1	0.51(0.23–1.14)	.1	0.87(0.39–1.95)	.741
T2	1.04(0.46–2.31)	.93	1.65(0.74–3.69)	.223
T3	1.42(0.64–3.18)	.39	2.04(0.91–4.59)	.083
T4	3.12(1.39–6.98)	<.01	3.61(1.61–8.11)	<.01
**N_stage**				
N0	Reference	NA	Reference	NA
N1	1.53(1.42–1.67)	<.001	1.48(1.35–1.61)	<.001
N2	2.83(2.55–3.15)	<.001	2.68(2.39–3.00)	<.001
N3	3.66(3.25–4.12)	<.001	3.06(2.68–3.48)	<.001
**Subtype**				
HR+/HER2+	Reference	NA	Reference	NA
HR–/HER2+	1.47(1.37–1.57)	<.001	0.83(0.65–1.06)	.14
**ER**				
Positive	Reference	NA	Reference	NA
Negative	1.49(1.39–1.59)	<.001	1.36(1.09–1.71)	<.01
**PR**				
Positive	Reference	NA	Reference	NA
Negative	1.48(1.38–1.58)	<.001	1.30(1.19–1.43)	<.001
**Surgery**				
Yes	Reference	NA	Reference	NA
No	3.87(3.49–4.29)	<.001	2.31(2.07–2.58)	<.001
**Radiation**				
Yes	Reference	NA	Reference	NA
No	1.57(1.47–1.69)	<.001	1.30(1.20–1.40)	<.001
**Chemotherapy**				
Yes	Reference	NA	Reference	NA
No	2.28(2.13–2.44)	<.001	3.03(2.80–3.27)	<.001

CI = confidence interval, ER = estrogen receptor, HER2 = human epidermal growth factor receptor-2, HER2+ = human epidermal growth factor receptor-2 positive, HR– = hormone-receptor-negative, HR+ = hormone-receptor-positive, HRs = hazard ratios, LIQ = lower inner quadrant, LOQ = lower outer quadrant, PR = progesterone receptor, UOQ = upper outer quadrant, UIQ = upper inner quadrant.

her2 = human epidermal growth factor receptor-2, OS = overall survival.

### 3.3. Nomogram validation

**Figure 2.**
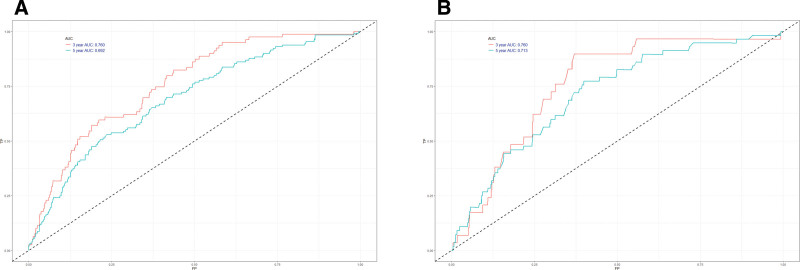
ROC curve analysis to predict 3- and 5- year OS rates of female patients suffering non-metastatic her2 positive breast cancer. (A) ROC curve for the training cohort. (B) ROC curve for the external validation cohort.

**Figure 3.**
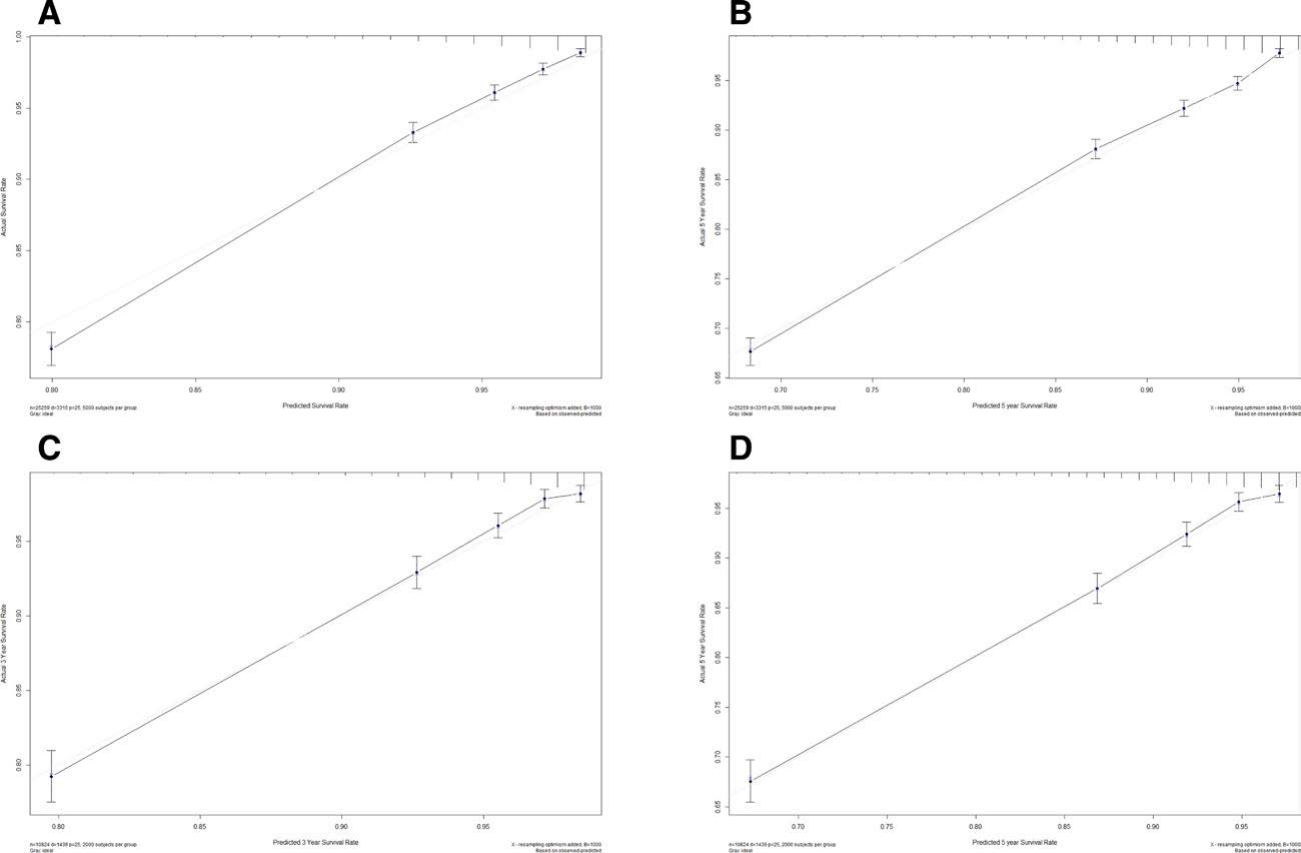
Calibration plots for predicting 3- and 5- year OS rates in female patients suffering non-metastatic her2 positive breast cancer. (A) Calibration plot of the 3-year training cohort. (B) Calibration plot of the 5-year for training cohort. (C) Calibration plot of the 3-year for the external validation cohort. (D) Calibration plot of the 5-year for the external validation cohort. The x-axis represents the predicted OS in female patients suffering from non-metastatic her2-positive breast cancer. The y-axis represents the actual OS in female patients suffering from non-metastatic her2 positive breast cancer. The diagonal dotted line stands for a perfect prediction using an ideal model. We drew the solid line to represent the performance of the nomogram, of which the closer fit to the diagonal dotted line represents the better prediction of the nomogram.

The training cohort was employed to internally validate the nomogram. Harrell C-index was 0.764 (95% confidence interval: 0.756–0.772) in the training set which indicates discrimination ability. To be similar, Harrell C-index was 0.757 (95% confidence interval: 0.743–0.771) in the external validation set. Moreover, the 3- and 5-year AUC values of the training set were 0.760 and 0.692, corresponding to 0.760 and 0.713 in the validation set (Fig. 2). According to these outcomes, the OS can be accurately predicted by the nomogram. The nomogram performance was evaluated by using the internal and external calibration plots. A worse prognosis was caused by the higher total points according to the sum of the appointed number of points for every recognized element in the nomogram. According to Figure 3, the calibration plots for predicting the 3- and 5-year OS in both the training excellently agreed with those in validation sets.

AUC = the area under the ROC curve, FP = false-positive rate, her2 = human epidermal growth factor receptor-2, OS = overall survival, ROC = receiver operating characteristic curve, TP = true positive rate.

her2 = human epidermal growth factor receptor-2, OS = overall survival.

## 4. Discussion

According to univariable and multivariable Cox proportion hazards regression, we identified the correlated prognostic elements in the OS rate of female Her-2 positive breast cancer patients without metastasis. And the factors included age at diagnosis, marital status, race, site, T stage, N stage, PR status, ER status, surgery, radiation, and chemotherapy. We excluded metastatic disease as its treatment is generally delivered with palliative rather than curative intent. Meanwhile, we used the SEER database with a mean follow-up set up nomogram quantificationally predicting the 3- and 5-year OS rates by patient-associated and tumor-associated elements. Our findings can inform preventive and therapeutic strategies aimed at improving survival for these women.

Age at diagnosis is related to breast cancer survival. It is reported that approximately 5% of breast cancer are diagnosed in females who are younger than 40 years of age.^[9]^ In fact, during the review time almost 90% of patients more youthful than 40 years old died from their breast cancer, contrasted and just 49% of patients 40 years old and more seasoned. Several studies had shown that younger breast cancers often exhibited more aggressive biological characteristics, such as estrogen receptor-negative (ER–) and HER2 positive tumor, high grade and are more prone to lymph node metastasis, resulting in higher recurrence and mortality rates.^[10–14]^ Thus, in this study, we used an age cutoff of 40 years to perform the relationship between age and prognosis in her2 positive breast cancer patients. In our study, we noted that elderly women experience poorer outcomes, which conformed to past publications.^[15–17]^ The reasons for the worse results noted among older patients were multifaceted. One important factor is that older patients tend to nonstandard treatment because of lower tolerance to surgery, chemotherapy, and radiotherapy.^[18,19]^ In our analysis, marital status has a higher hazard ratio than age, race, receipt of radiation, grade, T stage (1–2), and N1 disease. And married patients show a better prognosis than others including single, divorced, widowed, separated, unmarried, or domestic partner in our manuscript. Marital status and ethnicity as the most important forms of social relations have been suggested as predictive factors for breast cancer survival. Married patients were found to have more emotional and financial support. They could be diagnosed at an earlier stage, received proper treatments, and prolonged their overall survival.^[20–22]^ According to our findings, perhaps more attention should be paid to psychological and social support during the treatment of breast cancer patients.

Contemporarily, many clinicopathological characteristics are taken into consideration for prognosis in breast cancer patients, such as site, grade, T stage, N stage, breast cancer sub-type, ER status, PR status. Previous studies displayed that anatomic site was a great independent element. Our study finds agreement with others that breast tumors in the lower outer quadrant (LOQ) have the best prognosis,^[23]^ although tumors in the upper outer quadrant (UOQ) are generally considered to have the best prognosis.^[24,25]^ Anatomical differences can significantly affect the development of tumor metastasis, which is very important for the prognosis of breast cancer. Metastases to the internal mammary nodes are difficult to detect on imaging, leading to inadequate diagnosis, and treatment.^[26,27]^ Compared to more medial tumors, outer regions tumor showed better prognosis due to the lymphatic involvement was easily detected and complete surgical management.^[28]^ Besides, we find laterality was not regarded as a prognostic factor on OS rates of female Her-2 positive breast cancer without metastasis. However, B. Karatas F et al demonstrated left laterality was an independent prognostic factor for metastasis in N3 stage breast cancer.^[29]^ And it is reported that left-side radiotherapy was associated with increased cardiac mortality.^[30]^ The underlying mechanism of laterality and site on breast cancer outcomes need to be further explored. ER (+) and progesterone receptor-positive (PR (+)) have been regarded as protective elements for the prognosis of breast cancer in most past research.^[31,32]^ It is argued that hormone therapy is effective for hormone-receptor-positive (HR+) patients, which provides broader therapeutic approaches for her2 positive patients. Control trials have reported that despite over-expression of the HER2 oncogene, hormone receptor state is still a great determinant of disease result, with more recurrences and deaths among females with the hormone-receptor-negative disease even after 11 years’ median follow-up.^[33]^ Interestingly, we found that ER (+) and PR (+) exhibited higher hazard ratio, yet HR+ subtypes showed no statistical significance in hazard ratio. By now, data on HR subtypes in her2 positive breast cancer patients are limited. Early studies reported that the HR+/HER2+ subtype was associated with better prognoses of breast cancer patients than the hormone-receptor-negative (HR–)/HER2+ subtype.^[34,35]^ On the contrary, Bae et al^[36]^ found no difference in OS of her2+ breast cancer patients in the comparison among four subtypes (estrogen receptor-positive [ER+]/[PR+], ER–/PR+, ER+/progesterone receptor-negative [PR–], ER–/PR–). Our study showed similar results. The authors showed that single HR+ subtypes (ER–/PR+, ER+/PR–) were not significantly associated with OS in HER2-positive breast cancer. These results indicated that HER2 expression and anti-her2 therapy may be more significant prognostic factors than single HR+ expression in HER2+ breast cancer.

AJCC T stage, AJCC N stage, were recognized prognostic elements for breast cancer,^[37,38]^ and our study showed the same results. Using the 7th edition of the AJCC category to predict breast cancer prognosis is a traditional and classical protocol. The TNM grading system has proven to be an excellent tool for predicting breast cancer prognosis and guiding therapeutic selection worldwide. In terms of therapy, the approaches for her2 positive breast cancer include surgery, chemotherapy, radiotherapy, endocrine therapy, and anti-HER2 therapy. The wide application of the mentioned made contributions to reducing locoregional and distant recurrence, which can absolutely benefit the patients. In a meta-analysis by the EBCTCG, post-mastectomy radiotherapy for patients with axillary lymph nodes decreased the 10-year first recurrence rate by 10.6%, resulting in an 8.1% decrease in breast cancer mortality after 20 years.^[39]^ The anti-HER2 therapy (mainly trastuzumab and pertuzumab) with chemotherapy led to dramatic improvements in the survival of patients suffering from HER2 positive breast cancer.^[40]^

Luo et al^[41]^ explored 1304 consecutive patients suffering from non-metastatic HER2 positive breast cancer and identified several independent prognostic elements to set up a nomogram. The authors utilized their clinical database as the training set, which did provide treatment details, and then capitalized on the SEER confirmation. But in our manuscript, we selected a huge patient population from the SEER database to construct a nomogram, which also displayed an excellent discrimination power to predict prognosis. The study of Luo et al and ours show consistency and support each other, making the results more convincing.

Although the nomogram displayed excellent discrimination and performance, our research contains certain restrictions. Firstly, other factors with certain guidance indications were not available from the SEER database, including the presence of surgical margin state, levels of Ki-67, and kind of chemotherapy. Secondly, the administration of anti-HER2 therapy and hormonal therapy were much beyond our accessibility. Thirdly, some variables and categorizing them as “others” might result in data bias. And it is required that the nomograms shall be the external validation by prospective cohort before the application to clinical practice since the study relies on historical information.

## 5. Conclusion

In conclusion, demographic and clinicopathological features were incorporated from a large population-based cohort to set up an efficient nomogram for predicting the prognosis of female patients suffering from non-metastatic HER2 positive breast cancer. With regard to the nomogram, clinicians can more accurately forecast individual overall mortality within 3 or 5 years, which will lay a foundation for subsequent administration methods.

## Acknowledgments

We are very grateful to the SEER program for approving the registration and to the SEER database.

## Authors contributions

Jiangwen Wu, Yu Xiao, and Pengcheng Zhang designed the research. Jiangwen Wu, Yu Xiao, and Zhaomin Xie performed the research and analyzed the results. Jiangwen Wu, Bingbing Wang, and Yu Xiao wrote the paper. All authors edited the manuscript and provided critical comments. All authors read and approved the final manuscript.

Conceptualization: Pengcheng Zhang.

Data curation: Yu Xiao.

Formal analysis: Zhaomin Xie, Yu Xiao.

Investigation: Zhaomin Xie, Bingbing Wang.

Methodology: Jiangwen Wu, Bingbing Wang.

Project administration: Pengcheng Zhang.

Resources: Zhaomin Xie.

Software: Zhaomin Xie, Yu Xiao.

Supervision: Yu Xiao, Bingbing Wang, Pengcheng Zhang.

Validation: Yu Xiao.

Writing – original draft: Jiangwen Wu, Pengcheng Zhang.

Writing – review & editing: Jiangwen Wu, Pengcheng Zhang.

## References

[1] Siegel RL Miller KD Fuchs HE. Cancer statistics, 2021. CA Cancer J Clin. 2021;71:7–33. 33433946 10.3322/caac.21654

[2] Bray F Ferlay J Soerjomataram I. Global cancer statistics 2018: GLOBOCAN estimates of incidence and mortality worldwide for 36 cancers in 185 countries. CA Cancer J Clin. 2018;68:394–424. 30207593 10.3322/caac.21492

[3] Yarden Y Sliwkowski MX. Untangling the ErbB signalling network. Nat Rev Mol Cell Biol. 2001;2:127–37. 11252954 10.1038/35052073

[4] Wolff AC Hammond MEH Hicks DG. Recommendations for human epidermal growth factor receptor 2 testing in breast cancer: American Society of Clinical Oncology/College of American Pathologists clinical practice guideline update. Arch Pathol Lab Med. 2014;138:241–56. 24099077 10.5858/arpa.2013-0953-SA PMC4086638

[5] Murphy BL Day CN Hoskin TL. Neoadjuvant chemotherapy use in breast cancer is greatest in excellent responders: triple-negative and HER2+ subtypes. Ann Surg Oncol. 2018;25:2241–8. 29786125 10.1245/s10434-018-6531-5

[6] Lewis Phillips GD Li G Dugger DL. Targeting HER2-positive breast cancer with trastuzumab-DM1, an antibody-cytotoxic drug conjugate. Cancer Res. 2008;68:9280–90. 19010901 10.1158/0008-5472.CAN-08-1776

[7] Chia S Norris B Speers C. Human epidermal growth factor receptor 2 overexpression as a prognostic factor in a large tissue microarray series of node-negative breast cancers. Journal of Clinical Oncology. 2008;26:5697–704. 19001334 10.1200/JCO.2007.15.8659

[8] Balachandran VP Gonen M Smith JJ. Nomograms in oncology: more than meets the eye. Lancet Oncol. 2015;16:e173–80. 25846097 10.1016/S1470-2045(14)71116-7 PMC4465353

[9] DeSantis CE Ma J Gaudet MM. Breast cancer statistics, 2019. CA Cancer J Clin. 2019;69:438–51. 31577379 10.3322/caac.21583

[10] Chung M Chang HR Bland KI. Younger women with breast carcinoma have a poorer prognosis than older women. Cancer. 1996;77:97–103. 8630946 10.1002/(SICI)1097-0142(19960101)77:1<97::AID-CNCR16>3.0.CO;2-3

[11] Adami H-O Malker B Holmberg L. The relation between survival and age at diagnosis in breast cancer. N Engl J Med. 1986;315:559–63. 3736639 10.1056/NEJM198608283150906

[12] El Saghir NS Seoud M Khalil MK. Effects of young age at presentation on survival in breast cancer. BMC Cancer. 2006;6:1–8. 16857060 10.1186/1471-2407-6-194 PMC1555600

[13] Nixon AJ Neuberg D Hayes DF. Relationship of patient age to pathologic features of the tumor and prognosis for patients with stage I or II breast cancer. J Clin Oncol. 1994;12:888–94. 8164038 10.1200/JCO.1994.12.5.888

[14] Richards M Gregory W Smith P. Age as prognostic factor in premenopausal breast cancer. Lancet (British edition). 1993;341:1484–5. 10.1016/0140-6736(93)90932-7 8099185

[15] Freedman RA Keating NL Lin NU. Breast cancer-specific survival by age: worse outcomes for the oldest patients. Cancer. 2018;124:2184–91. 29499074 10.1002/cncr.31308 PMC5935594

[16] Gnerlich JL Deshpande AD Jeffe DB. Elevated breast cancer mortality in women younger than age 40 years compared with older women is attr ibuted to poorer survival in early-stage disease. J Am Coll Surg. 2009;208:341–7. 19317994 10.1016/j.jamcollsurg.2008.12.001 PMC3262236

[17] Chen HL Zhou MQ Tian W. Effect of age on breast cancer patient prognoses: a population-based study using the SEER 18 database. PLoS One. 2016;11:e0165409. 27798652 10.1371/journal.pone.0165409 PMC5087840

[18] Owusu C Lash TL Silliman RA. Effect of undertreatment on the disparity in age-related breast cancer-specific survival among older women. Breast Cancer Res Treat. 2007;102:227–36. 17004115 10.1007/s10549-006-9321-x

[19] Derks MGM Bastiaannet E van de Water W. Impact of age on breast cancer mortality and competing causes of death at 10 years follow-up in the adjuvant TEAM trial. Eur J Cancer. 2018;99:1–8. 29885375 10.1016/j.ejca.2018.04.009

[20] Woods L Rachet B Coleman M. Origins of socio-economic inequalities in cancer survival: a review. Ann Oncol. 2006;17:5–19. 16143594 10.1093/annonc/mdj007

[21] Osborne C Ostir GV Du X. The influence of marital status on the stage at diagnosis, treatment, and survival of older women with breast cancer. Breast Cancer Res Treat. 2005;93:41–7. 16184457 10.1007/s10549-005-3702-4

[22] Hinyard L Wirth LS Clancy JM. The effect of marital status on breast cancer-related outcomes in women under 65: a SEER database analysis. Breast. 2017;32:13–7. 28012410 10.1016/j.breast.2016.12.008

[23] Siotos C McColl M Psoter K. Tumor site and breast cancer prognosis. Clin Breast Cancer. 2018;18:e1045–52. 29941391 10.1016/j.clbc.2018.05.007

[24] Jing B Ke-Da Y Yi-Zhou J. The effect of laterality and primary tumor site on cancer-specific mortality in breast cancer: a SEER population-based study. PLoS One. 2014;9:e94815. 24740002 10.1371/journal.pone.0094815 PMC3989248

[25] Sohn VY Arthurs ZM Sebesta JA. Primary tumor location impacts breast cancer survival. Am J Surg. 2008;195:641–4. 18424280 10.1016/j.amjsurg.2007.12.039

[26] Colleoni M. Site of primary tumor has a prognostic role in operable breast cancer: the international breast cancer study group experience. J Clin Oncol Off J Am Soc Clin Oncol. 2005;23:1390. 10.1200/JCO.2005.06.052 15735115

[27] Hwang K-T Kim J Kim E-K. Poor prognosis of lower inner quadrant in lymph node–negative breast cancer patients who received no chemotherapy: a study based on nationwide Korean breast cancer registry database. Clin Breast Cancer. 2017;17:e169–84. 28169145 10.1016/j.clbc.2016.12.011

[28] Kroman N Wohlfahrt J Mouridsen HT. Influence of tumor location on breast cancer prognosis. Int J Cancer. 2003;105:542–5. 12712447 10.1002/ijc.11116

[29] Karatas F Sahin S Erdem GU. Left laterality is an independent prognostic factor for metastasis in N3 stage breast cancer. J Buon Off J Balkan Union Oncol. 2016;21:851. 27685905

[30] Darby SC Mcgale P Taylor CW. Long-term mortality from heart disease and lung cancer after radiotherapy for early breast cancer: prospective cohort study of about 300,000 women in US SEER cancer registries. Lancet Oncol. 2005;6:557–65. 16054566 10.1016/S1470-2045(05)70251-5

[31] Zhai Z Zhang F Zheng Y. Effects of marital status on breast cancer survival by age, race, and hormone receptor status: a population-based Study. Cancer Med. 2019;8:4906–17. 31267686 10.1002/cam4.2352 PMC6712463

[32] Johansson ALV Trewin CB Fredriksson I. In modern times, how important are breast cancer stage, grade and receptor subtype for survival: a population-based cohort study. Breast Cancer Res. 2021;23:17. 33526044 10.1186/s13058-021-01393-z PMC7852363

[33] Cameron D Piccart-Gebhart MJ Gelber RD. 11 years’ follow-up of trastuzumab after adjuvant chemotherapy in HER2-positive early breast cancer: final analysis of the HERceptin Adjuvant (HERA) trial. Lancet. 2017;389:1195–205. 28215665 10.1016/S0140-6736(16)32616-2 PMC5465633

[34] Untch M Gelber R Jackisch C. Estimating the magnitude of trastuzumab effects within patient subgroups in the HERA trial. Ann Oncol. 2008;19:1090–6. 18296421 10.1093/annonc/mdn005

[35] Hwang K-T Kim J Jung J. Impact of breast cancer subtypes on prognosis of women with operable invasive breast cancer: a population-based study using SEER DatabaseBreast cancer subtype and prognosis. Clin Cancer Res. 2019;25:1970–9. 30559169 10.1158/1078-0432.CCR-18-2782

[36] Bae SY Kim S Lee JH. Poor prognosis of single hormone receptor-positive breast cancer: similar outcome as triple-negative breast cancer. BMC Cancer. 2015;15:1–9. 25880075 10.1186/s12885-015-1121-4 PMC4396721

[37] Group EBCTC. Comparisons between different polychemotherapy regimens for early breast cancer: meta-analyses of long-term outcome among 100 000 women in 123 randomised trials. Lancet. 2012;379:432–44. 22152853 10.1016/S0140-6736(11)61625-5 PMC3273723

[38] Liu Y He M Zuo W-J. Tumor size still impacts prognosis in breast cancer with extensive nodal involvement. Front Oncol. 2021;11:585613. 33898305 10.3389/fonc.2021.585613 PMC8064390

[39] Ebctcg McGale P Taylor C. Effect of radiotherapy after mastectomy and axillary surgery on 10-year recurrence and 20-year breast cancer mortality: meta-analysis of individual patient data for 8135 women in 22 randomised trials. Lancet. 2014;383:2127–35. 24656685 10.1016/S0140-6736(14)60488-8 PMC5015598

[40] Loibl S Gianni L. HER2-positive breast cancer. Lancet. 2017;389:2415–29. 27939064 10.1016/S0140-6736(16)32417-5

[41] Luo C Zhong X Wang Z. Prognostic nomogram for patients with non-metastatic HER2 positive breast cancer in a prospective cohort. Int J Biol Markers. 2019;34:41–6. 30852974 10.1177/1724600818824786

